# Impact of Grape Seed Powder and Black Tea Brew on Lipid Digestion—An In Vitro Co-Digestion Study with Real Foods

**DOI:** 10.3390/nu15102395

**Published:** 2023-05-20

**Authors:** Judit Tormási, László Abrankó

**Affiliations:** Department of Food Chemistry and Analytical Chemistry, Institute of Food Science and Technology, Hungarian University of Agriculture and Life Sciences (MATE), Villányi Street 29-43, 1118 Budapest, Hungary

**Keywords:** lipid digestibility, milk fat, beef fat, gastric lipase, grape seed powder, black tea, bioaccessibility

## Abstract

Effects of two foods with bioactive constituents (black tea brew, BTB and grape seed powder, GSP) on lipid digestibility was studied. Lipolysis inhibitory effect of these foods was examined using two test foods (cream and baked beef) with highly different fatty acid (FA) composition. Digestion simulations were performed either using both gastric and pancreatic lipase, or only with pancreatic lipase according to the Infogest protocol. Lipid digestibility was assessed based on the bioaccessible FAs. Results showed the triacylglycerols containing short- and medium-chain FAs (SCFA and MCFA) are non-preferred substrates for pancreatic lipase; however, this is not characteristic for GL. Our findings suggest that both GSP and BTB primarily affect the lipolysis of SCFAs and MCFAs, because the dispreference of pancreatic lipase towards these substrates was further enhanced as a result of co-digestion. Interestingly, GSP and BTB similarly resulted in significant decrease in lipolysis for cream (containing milk fat having a diverse FA profile), whereas they were ineffective in influencing the digestion of beef fat, having simpler FA profile. It highlights that the characteristics of the dietary fat source of a meal can be a key determinant on the observed extent of lipolysis when co-digested with foods with bioactive constituents.

## 1. Introduction

Obesity and associated diseases such as metabolic syndrome, cardiovascular disease, type 2 diabetes, are unarguably among the most important health concerns [1,2,3,4]. The imbalance between the calories consumed with food and the energy expenditure of the body plays an important role in the development of obesity [5]. The contributions of both total dietary fat intake and percentage of energy intake from fat to obesity were shown by many studies [6,7]. However, others also indicated that this observation cannot always be confirmed at the population level [8] or divergencies were found for some sociodemographic groups [9]. These findings may support the rather fatty acid-dependent than generic associations between dietary fat intake and body weight, which is also shown in a number of studies [10,11].

Dietary fats—mainly comprised of triacylglycerols (TAGs)—are at least partly hydrolyze to free fatty acids (FFAs) via enzymatic hydrolysis during human digestion. At the end of lipid digestion, FFAs and monoacylglycerols (MAGs) are counted as bioaccessible molecules from which after trough passive or active transport part are transferred into the bloodstream, providing bioavailable molecules for further mechanisms [12,13,14]. The two main enzymes guiding the breakdown of food lipids are gastric lipase (GL) and pancreatic lipase (PL). The first step takes place in the stomach and is performed by GL; however, the majority of the lipid digestion by PL in the duodenum [15]. In general, GL contributes to approximately 5–40% of overall lipid digestion [16]. In addition, GL also serves as a catalyst for further lipid digestion, i.e., pre-digestion and emulsification enhances the efficacy of pancreatic lipase digestion [17]. Following the gastric pre-digestion, the food bolus enters the duodenum where PL continues the lipolysis. In total, more than 50% of the overall lipolysis is performed by PL in the small intestine. TAG hydrolysis—both performed by GL or PL—results in release of FFAs and partly hydrolyzed products such as MAGs, and diacylglycerols (DAG). It is important to note that unhydrolyzed TAGs are also present during the digestion process.

Among the many approaches applied in clinical obesity therapy, drug treatments using lipase inhibitors became an important means of clinical obesity treatment [18]. By restricting the liberation of fatty acids (FA), the bioaccessibility of dietary lipids can be modulated [19]. In Europe and in the US, currently, the only authorized medicine for lipase inhibition used in clinical treatment is Orlistat (lipstatin, Xenical^®^).

Alternatively, natural lipase inhibitors present in our food commodities are consumed over longer periods of time as a part of our everyday diet, and they can also play an important role in reduction in dietary lipid bioaccessibility and, consequently, preventing obesity [19,20,21]. Promising examples of foods with lipase inhibitory effect were reported spices, e.g., rosemary [22] and sage [23], industrial waste products such as pomegranate leaf [24] or grape seed [25,26], and commonly consumed beverages such as tea cultivars [27,28]. 

Wine production is one of the most important agricultural activities in the world and causes the generation of a large number of by-products, including grape skins, -seeds, and -stems [29]. Wine by-products have high potential as food ingredients, since they facilitate increased sustainability increase in the wine industry by reusing a product that is usually considered waste [30]. Therefore, further utilization of by-products can be of great importance, and a great deal of research effort is being devoted to testing the putative beneficial effects of grape parts [31]. Both grape seed [25] and grape skin [32] extract showed potential pancreatic lipase inhibitory effect according to in vitro enzyme assays using model substrates. Lipolysis inhibitory effect is often attributed to a variety of polyphenolic compounds, e.g., proanthocyanidins or condensed tannins, found in solid parts of grape (seeds and skins) [26,33,34,35,36]. Lipid profile of grapeseed was also studied and it was found that most abundant fatty acids are linoleic acid (C18:2 ω-6), oleic acid (C18:1), palmitic acid (C16:0), and stearic acid (C18:0), which represent about 98% of the total fatty acids [37].

Tea (*Camellia sinensis*) is one of the most frequently consumed beverages [38]. The health benefits of several types of tea (white [39], oolong [27], Pu-erh [28], green [38] and black [40,41]) were studied, which all are associated with potent lipase inhibitory properties. and these effects are closely related to the structure and composition of polyphenols. Catechins, which, during the fermentation process, are converted to theaflavins and large polymeric compounds, such as thearubigins, are the characteristic polyphenols in black tea brew [42]. 

It should be noted that the mechanism of action of different agents resulting in a reduction in lipid bioaccessibility can be highly different. Some of them—such as Orlistat—can bind directly to the active site of PL, whereas natural compounds are more likely to act indirectly, e.g., by interacting the oil–water interface [43]. Moreover, the role of polyphenols in terms of synergisms with other non-polyphenol constituents and their effect on the interface microenvironment is still inconclusive. It suggests that despite the available evidence on the role of polyphenols in lipase inhibition, it may be misleading at this stage of our knowledge to attribute the effect of polyphenol-rich foods such as grape seed powder or tea brew on lipolysis exclusively to polyphenols. Therefore, it would be desirable to study the putative effects of such bioactive components of foods such as polyphenols in their natural matrix or as part of a meal, since beyond direct lipase inhibition, food composition and co-digestion of other foods are also considered as key determinants of the extent of lipolysis.

The effect of food matrix, lipid content and composition on bioaccessibility and bioavailability of bioactive substances using the Infogest in vitro digestion simulation were studied and reviewed [44,45,46]. These studies highlight the importance of the presence of the lipid molecules, the sufficient emulsification and lipid hydrolysis during digestion on the release of bioactive molecules. In addition, it was reported that oils with different composition might show different overall bioaccessibility in presence of the same bioactive molecule, such as β-carotene or eugenol [47,48]. However, these results are based on the use of model matrices and there is still a lack of studies providing data on lipid digestibility from real foods and on how it is affected by bioactive substances.

Although there are some exceptions where more complex digestion simulation is used [49], most studies still focus on use of enzyme assays with model substrates and the diversity in the lipid composition of consumed food commodities is often neglected. Based on the encouraging results of the enzyme assays on the influence of grape seed powder (GSP) and black tea brew (BTB) on lipid digestion, the primary aim of this work was to evaluate the impact of these two selected foods on lipid digestion when co-digested with real foods with diverse lipid profile, under conditions that closely simulates human digestion [50].

## 2. Materials and Methods

### 2.1. Materials

For digestion protocol: porcine α-amylase (EC 3.2.1.1), porcine pepsin (EC 3.4.23.1), porcine pancreatin (8 × USP; P7545) and porcine bile extract (E.C. 3.1.1.3) was purchased from Merck/Sigma-Aldrich, St. Louis, MO, USA. Rabbit gastric lipase (RGE15) was purchased from Lipolytech Ltd., Marseille, France. FAME mixture (CRM47885) and internals standards; glyceryl trinonadecanoate (C19:0 TAG; >99%), methyl nonadecanoate (C19:0 ME, analytical standard), glyceryl triheptadecanoate (C17:0 TAG) and heptadecanoic acid (C17:0 FFA) were also obtained from Merck/Sigma-Aldrich. L-serine standard was purchased from Reanal Ltd., Budapest, Hungary Reagents and solvents were of analytical purity. Chloroform was purchased from Carlo Erba Reagents, Emmendingen, Germany (for analysis, stabilized with ethanol), methanol (for HPLC, LC-MS grade) and hydrochloric acid (37%) from VWR International, Radnor, PA, USA, isooctane from Fisher Scientific, Waltham, MA, USA (>95%). High purity water (>18 MΩcm^−1^) was prepared by a Millipore Elix Essential 3 UV Water Purification System (Merck-Millipore, Burlington, MA, USA). Orlistat (>98% solid) were purchased from Sigma-Aldrich.

### 2.2. Sample Preparation

Substrate test foods, ground beef (20% fat content) and cream (30% fat content) were purchased from local supermarkets. Ground beef was baked before digestion experiments for 20 min in a 200 °C oven and it was then homogenized in a household meat grinder three times. Baked beef (BB) samples were stored at −80 °C and thawed before experiments. Cream (C) was always purchased fresh and used right after opening and thorough mixing on room temperature. These substrate test foods were selected as representatives of natural dietary lipids sources to gain information on the effect of fat-free foods; grape seed powder and black tea on lipid digestion.

Grape seed powder (GSP) was provided by Bock Vineyard Ltd. (Villány, Hungary), and the powder was added to substrate test foods (BB and C) in multiple concentrations before digestion experiments, separately. Black tea (Himalayan Spring FF 2022 No. 601) with high tannin content were bought commercially. Tannin content (163 mg/g tea leaf) was measured according to the method provided for determination of tannin content of tea species [51]. The same extract as to determine tannin content was used in digestion experiments, as black tea brew (BTB). BTB was added to digestion simulations test foods (baked beef and cream) in multiple concentrations before the experiments.

Determination of tannin content was as follows. Into 50-mL round bottom flasks, 0.2000 g of black tea leaves were measured and 50 mL distilled water was added. Water was heated on sand for 1 h (with water cooler system attached to prevent evaporation). After cooling, extracts were sieved on paper sieve and collected filtrate was completed to 50 mL. One part of the extract was used to determine tannin content, the rest was stored in −80 °C until use in digestion experiments. For measuring tannin content, extract was heated until boiling and 30 mL 5 *w*/*v*% copper–acetate solution was added to precipitate tannin content. Extract was sieved and pellets were washed until no residual copper was left. Pellets were cremated, rewetted with nitric acid and re-cremated. Tannin content was determined by weight.

### 2.3. Determination of Fat Content of Test Foods

For evaluating total fat content of baked beef, the standard method available for meat and meat products was used [52] based on Soxhlet extraction with petroleum ether, and gravimetric determination. Total fat content of cream was determined according to ISO 2450:2009 [53] using cold, organic extraction with the mixture of diethyl- and petroleum ether, and gravimetric determination. Total fat contents of test foods were given in g fat/100 g product. Fatty acid composition was determined from the extracted fats with the GC-FID method described in Tormási and Abrankó [54].

### 2.4. In Vitro Digestion Simulation

Digestion simulations were made according to Infogest internationally acknowledged consensus in vitro digestion simulation protocol [50]. The original 2014 version of this protocol did not include gastric lipase, whereas the most recent version suggests to also use gastric lipase (as a part of rabbit gastric extract) when lipid digestion is to be studied. To better understand the synergistic effect of GL in the overall lipid digestions by performing pre-digestion, both versions of the protocol were used. In the version hereafter called GL + PL, lipolytic enzymes gastric lipase (as a part of rabbit gastric extract (RGE) also containing pepsin) and pancreatic lipase (as a part of pancreatin enzyme complex) were used. In the second version, hereafter called PL, only pancreatic lipase (as a part of pancreatin enzyme complex) was used.

Simulated stock electrolyte solutions (simulated salivary fluid; SSF, simulated gastric fluid; SGF, simulated intestinal fluid; SIF) were made beforehand, and pH was adjusted to 7 (oral), 3 (gastric) and 7 (small intestinal), respectively. Enzyme activities were measured via methods described in Brodkorb et al., and enzyme stock solutions were made right before use. Required volume of 6 M HCl and 1 M NaOH to keep pH was determined before each digestion experiment with a “pH test” using the same amounts of samples and solvents only without enzymes.

For oral phase, 3.5 mL of SSF (tempered to 37 °C), 25 μL of 0.3 M CaCl_2_, 0.5 mL of amylase solution (1500 U/mL in SSF) and 0.975 mL of distilled water was added. Homogenized samples were incubated in an overhead shaker (Heidolph Reax 2, Heidolph Instruments, Schwabach, Germany) fitted inside a preheated drying cabinet (Memmert UNE300, Memmert GmbH, Schwabach, Germany) for 2 min at 37 °C. In the gastric phase, 6.4 mL of SGF (tempered to 37 °C), 5 μL of 0.3 M CaCl_2_, required volume of 6 M HCl, 1.6 mL of pepsin solution (25,000 U/mL in SGF) and 1.945 mL of water was added, and the mixture was incubated in the overhead shaker at 37 °C for 2 h. In the case of the GL + PL digestion, 1.6 mL of rabbit gastric extract (RGE, 750 U/mL in SGF) was added in the gastric phase and no pepsin was added. For the small intestine phase, 8.5 mL of SIF (tempered to 37 °C), 40 μL of 0.3 M CaCl_2_, 2.5 mL of bile extract solution (160 mM in SIF, tempered to 37 °C), 5 mL of pancreatin solution (800 U/mL in SIF), required volume of 1 M of NaOH and 3.86 mL of water was added, and mixture was incubated in the overhead shaker at 37 °C another 2 h. After small intestinal digestion phase was completed, weight of digests was measured.

In order to accurately characterize the effect of bioactive containing foods on lipid digestibility, fat content of the digests’ were set to 150 mg; therefore, sample weight varied according to fat content of the test foods. Control digestions (only substrate test foods) were carried out with 0.5 g cream and 0.9 g baked beef based on lipid content. Samples were measured into 50 mL centrifuge tubes and supplemented to 5 g with distilled water. In addition of lipid digestibility test of the two test foods, experiments were carried out by co-digestion of the test foods with two fat-free foods containing bioactive constituents (GSP, BTB). To determine the appropriate concentration, dose dependency tests of GSP and BTB were carried out with cream test food. To cream (0.5 g), BTB was added at 1:1, 1:2 and 1:3 (w) ratio or GSP was added at 5, 10 and 15 *w*/*w*%. Portions of GSP typically consumed are 5–10 g a day, which is in accordance with the used 5–15 *w*/*w*% concentration if consumed along with a tea drink or a meal containing 50–100 g meat. Portions of test foods with appropriate amounts of either GSP or BTB were homogenized and made up to 5 g in 50-mL test tubes before digestion simulation. Effect of GSP and BTB on BB were only tested at the most efficient level of addition, i.e., 5 *w*/*w*% for GSP and 1:2 ratio for BTB. All digestion experiments were conducted in triplicates. Blank digestions were also made for each triplicate using 5 g of distilled water as sample. Positive control for lipase inhibition studies were made with the addition of Orlistat (a lipase inhibitory drug), where 40 µL of 0.5 M Orlistat solution in DMSO was added to fully inhibit lipases (Results presented elsewhere [54]).

### 2.5. Assessment of Fatty Acid Specific Lipolysis—Bioaccessible Fatty Acid Content

Bioaccessible FA content and composition were measured according to Tormási and Abrankó, 2021 [54]. From digests, 5 mL samples were taken and all lipid compounds were extracted with the Bligh and Dyer method (1959) [55] after the addition of 250 μL of C19:0 TAG internal standard (ISTD) solution (1 mg/mL in CHCl_3_). After phase separation, from the lower phase (∑12.5 mL chloroform, containing fat components) 2 × 5 mL was pipetted into two round-bottom flasks and solvent was evaporated using a rotary evaporator. One aliquot was methylated according to the ISO 12966-2:2017 standard’s ‘general method’ to result the total fatty acid content (TFA method) [56]. The other 5-mL aliquot was trans methylated with the alkaline ‘fast method’ of the same ISO standard to obtain the esterified fatty acid content (EFA method). FA composition was determined with GC-FID (see chapter “Determination of fatty acid composition with GC-FID”). Total fat content (TFA) and esterified fat content (EFA) were used to determine amount of free fatty acids (FFA, Equation (1)), bioaccessibility or release ratio of each fatty acids (RR, Equation (2)) and bioaccessible fatty acid content in % (Equation (3)).

FFAi = TFAi − EFAi (1)

Bioaccessibility of FAi/Release Ratio = FFAi/TFAi (2)

Bioaccessible FA content [%] = (∑FFAi/∑TFAi) × 100 (3)

TFA_i_: total amount of individual FAs; EFA_i_: amount of the same FA in esterified form, FFA_i_: amount of the same free FA, ∑FFAi: sum of the amount of each individual FFAs and ∑TFAi: the sum of the amount of each individual FA all in the small intestinal digesta.

### 2.6. Statistics

Pairwise comparison was carried out in Microsoft Excel using Student’s *t* test. Significance was recognized at *p* < 0.05. Comparison of multiple samples was performed using ANOVA test in Microsoft Excel (LTSC MSO (16.0.14332.20279) 32-bit); however, where significant difference was found (*p* < 0.05), Tukey’s post hoc test was performed using IBM SPSS Statistics 25. Equality of variances were tested with Levene’s test (*p* > 0.05).

## 3. Results and Discussion

### 3.1. Substrate Test Foods with Different Fatty Acid and TAG Profile

The impact of black tea brew (BTB) and grape seed powder (GSP) on lipid digestion was studied using two substrate test foods: cream (C) and baked beef (BB). The lipid content of substrate test foods was considered as the substrate for lipolysis. Primarily, we wanted to choose real food samples as substrate test foods and, moreover, ones with highly different FA composition.

Milk fat (lipid source of cream) contains a diverse variety of FAs containing short chain fatty acids (SCFA), medium chain fatty acids (MCFA) and long chain fatty acids (LCFA).

**Figure 1:**
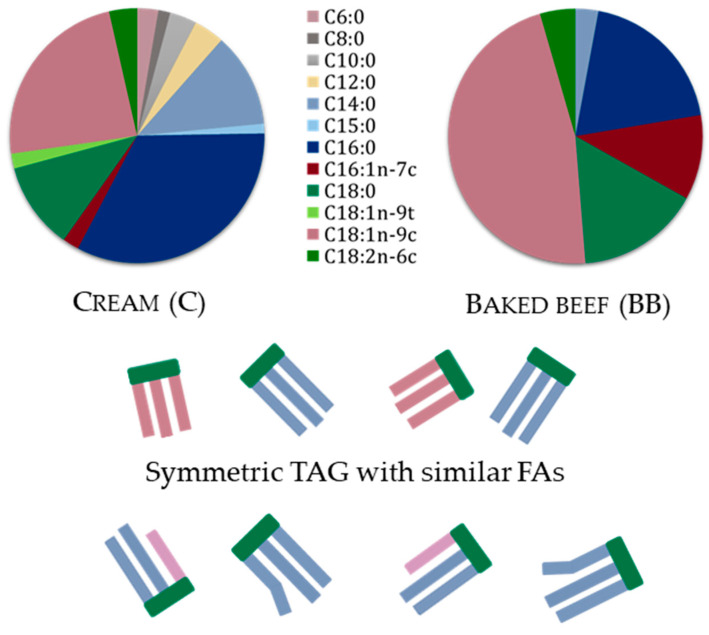
Fat content of test foods cream (C) and baked beef (BB) and illustration of most common TAG structures.

According to FA profiling carried out in this study, in the analyzed cream sample, the most abundant FAs were palmitic acid (C16:0: 33.3 ± 0.3%), oleic acid (C18:1n-9c: 24.5 ± 0.3%), stearic acid (C18:0: 11.9 ± 0.2%) and myristic acid (C14:0: 11.2 ± 0.1%). It also contained several FAs between 1 and 5 *w*/*w*% such as linoleic acid (C18:2n-6c: 4.1 ± 0.1%), capric acid (C10:0: 3.4 ± 0.04%), lauric acid (C12:0: 2.8 ± 0.2%), caproic acid (C6:0: 2.6 ± 0.03%), elaidic acid (C18:1n-9t: 2.4 ± 0.3%), palmitoleic acid (C16:1n-7c: 2.2 ± 0.03%), caprylic acid (C8:0: 1.5 ± 0.04%) and pentadecanoic acid (C15:0: 1.3 ± 0.02%) (Figure 1). The overwhelming majority (98.2 ± 0.1%) of the lipid content of the tested cream sample was made of twelve different FAs. The high degree of FA diversity measured in cream (milk fat) necessarily reflects in the types of TAGs as well. A mixture of TAGs with heterogeneous composition is characteristic of milk fat [57]. Short (C4-C8) chain FAs were largely located at the *sn*-3 position of TAGs next to MCFAs and LCFAs, whereas palmitic acid (C16:0) and oleic acid (C:18:1) were predominantly found in *sn*-2 position; however, the preferred position of the latter and also that of MCFAs depend on the actual size of the TAG [58]. Therefore, milk fat TAGs are considered to contain the most “asymmetric” TAGs among animal fats [59].

In contrast, the FA profile of beef fat was much simpler than that of milk fat. In the BB test food, only six of the FAs were above 1 *w*/*w*% and the majority of them were comprised of only five FAs, namely, oleic acid (C18:1n-9c: 44.9 ± 0.02%), palmitic acid (C16:0, 26.8 ± 0.06%), stearic acid (C18:0, 14.9 ± 0.08%), palmitoleic acid (C16:1n-7c; 5.0 ± 0.02%), myristic acid (C14:0: 2.6 ± 0.01%) and linoleic acid (C18:2n-6c, 2.1 ± 0.02%) (Figure 1). The three most abundant FAs (C18:1n-9c, C16:0 and C18:0) covered the 75.5 ± 0.21% of all FAs. Consequently, the structure of most beef TAGs was more symmetric than of cream TAGs [60].

### 3.2. Lipid Digestibility of Substrate Test Foods

Lipid digestibility of studied test foods was assessed based on measuring the extent of lipolysis. This was carried out according to our recently published method [54] based on the monitoring of the bioaccessible FFAs after in vitro digestion simulation.

The applied in vitro digestion simulation protocols allowed us to perform a mechanistic evaluation of the contribution of GL and PL to lipid digestibility of the test foods (cream and baked beef) and, later, of the effect of foods with bioactive constituents (GSP and BTB). Digestion simulations were performed either using both gastric lipase and pancreatic lipase (hereafter GL + PL) or only with pancreatic lipase (hereafter PL). After in vitro digestion simulation with both versions, the FFA content (Equation (1)) and RR (Equation (2)) of each individual FA were determined. Total lipid digestibility was assessed as the sum of individual FFAs (Equation (3)).

#### 3.2.1. Cream: Addition of Gastric Lipase Specifically Ameliorated Release of SFCAs and MCFAs

**Figure 2:**
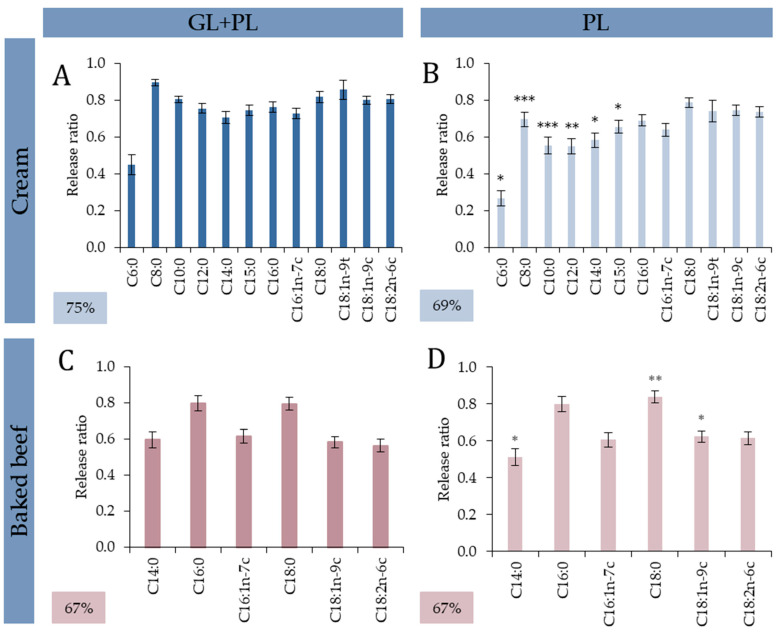
Bioaccessible fatty acid content of test foods (%; Equation (3)) and release ratios (Equation (2)) after Infogest digestion simulations (GL + PL and PL). (**A**): Cream GL + PL, (**B**): Cream PL; (**C**): Baked beef GL + PL, (**D**): Baked beef PL. Asterisk labels (*) show significant difference between release ratio of individual fatty acids after GL + PL and PL digestions (* *p* < 0.05; ** *p* < 0.01; *** *p* < 0.001).

Total lipid digestibility based on FA release of cream determined with GL + PL method was 77.1 ± 5.0% (RSD: 0.07). The RR of individual FAs slightly varied in the range of 0.70 and 0.82 (Figure 2A). Only one FA (C6:0) deviated from the rest, which showed relatively low digestibility (RR = 0.45) compared to the other FAs. These results show that the FA pattern of the food and the FFA profile of the digesta were similar; thus, no bias in either of the FA occurred during enzymatic hydrolysis of cream, when both lipolytic enzyme types were added.

To gain more insight to the different digestion mechanism of lipolysis with and without GL, digestibility of lipids was also measured after in vitro digestion simulation with PL alone. Compared to the results derived from GL + PL digestion, overall digestibility with PL digestion decreased by 11% to 69.2 ± 6.1%. This was in accordance with the literature consensus on the contribution of GL to overall lipid digestion, i.e., pre-digestion by GL adds between 5 and 40% to overall lipid digestion [16].

Nevertheless, the extent of lipolysis was markedly different for shorter chain FAs and longer chain FAs. Short and medium chain FAs showed RR from 0.55 to 0.65 and longer chained FAs showed RR from 0.69 to 0.73. The caproic acid (C6:0) were an outlier during PL digestions as well, RR value was only 0.27 to this FA. The addition of GL mostly affected the release of SCFAs and MCFAs during digestion simulation of cream, namely in the case of C6:0 (+40%), C8:0 (+22%), C10:0 (+31%), C12:0 (+27%), 14:0 (+17%) and C15:0 (+12%) (Figure 2A,B). This can be explained by the difference in the substrate preference of PL and GL. Both GL and PL enzymes have regiospecific preferences for FAs at the outer *sn*-1/3 positions [15,61,62,63], and it is also generally accepted that GL has a preference for the FAs in the *sn*-3 position in TAGs. However, a lesser preference over the *sn*-1 position was also reported. According to our results, the contribution of GL was most pronounced in the lipolysis of short (C4-C8) and medium (C10-C14) chain FAs. Short chain FAs are mainly located in the *sn*-3 position next to MCFAs (in *sn*-2) in smaller size TAGs [58], most probably forming asymmetric TAGs in milk fat. Our results also lend support to the assumption that that small-sized TAGs containing both SCFA and MCFA are non-preferred substrate types for PL. Consequently, PL alone is rather ineffective in the digestion of TAGs containing short and medium chain FAs and GL is of key importance in the lipolysis of such asymmetric TAGs of milk fat. The findings of Benito-Gallo et al. also suggest that PL showed the same selectivity towards the hydrolysis at positions *sn*-1 and *sn*-3 of the TAG, provided that the FA side chains were identical [64]. This further supports our observations regarding the key role of GL in the digestion of asymmetric TAGs of milk fat. Moreover, due to this shift in RR of these FAs with the addition of GL, the saturated FA level increased by 14% in the bioaccessible fraction of digested cream compared to just using PL.

#### 3.2.2. Baked Beef: No Additional Effect of GL without Diversity of TAGs

In the case of baked beef test food, total FA release and individual RR of FAs were also determined after in vitro digestion simulation. Interestingly, there was no difference between total digestibility measures determined after GL + PL and PL digestions. Overall, FA release was 67.7 ± 2.5% and 67.2 ± 1.6%, respectively (*p* = 0.742). In contrast, there were some differences between individual RRs of FAs. With the addition of GL, RR of C14:0 increased from 0.51 to 0.60 (*p* = 0.019) and RR of C18:0 and C18:2n-6c decreased slightly from 0.84 and 0.62 to 0.80 (*p* = 0.001) and 0.56 (*p* = 0.036), respectively (Figure 2C,D). However, the observed changes in the FA composition of the digesta caused by the difference in the RR of these FAs cancelled each other out; thus, no change was detectable in the overall digestibility of baked beef fat. The differences between the digestion simulation with and without GL showed that GL had a significant role in ameliorating and improving the release of certain FAs. Although the digestion simulation did not show variation between overall lipid digestibility of baked beef, the release of one MCFA (C14:0) increased significantly (+14%) and two others decreased (both LCFAs, C18:0 and C18:2n-6c; by 5–9%). It should be noted, however, that in the case of baked beef, FA composition of digesta differed from FA composition of the baked beef meal. It means, the relative amount of C14:0, C16:1n-7c, C18:1n-9c and C18:2n-6c decreased and C16:0 and C18:0 increased in the digesta compared to the test food; thus, SFA content of digesta also increased by 13.7%. Similarly, Hur et al. investigated the lipid digestibility of beef and, in line with our observations, they found that after in vitro digestion, the composition of digesta differed from initial FA composition of beef [65].

The results of our fat digestibility study on cream (milk fat) and baked beef showed the TAGs containing SCFAs and MCFAs were non-preferred substrate types for PL and, consequently, PL alone was rather ineffective in the digestion of such TAGs containing SCFAs and MCFAs. However, this specificity is not characteristic for GL. Thus, the presence of GL resulted in the increased release of SCFAs and MCFAs (C6:0–C15:0), especially if these FAs were part of asymmetric TAGs. This effect was more pronounced in milk fat containing products such as the analyzed cream. The results also showed that when there was no marked diversity in FA composition and, consequently, heterogeneity in the TAG structure is limited, the contribution of GL could be negligible such as in the case of baked beef, where overall fat digestibility remained the same. However, the contribution of GL to fat digestibility might be more complex in other food commodities. It was shown that the ability of GL to cleave the PL-not preferred FAs from *sn*-3 position would have additional benefits. The TAGs that are ineffectively digested by PL—such as presumably asymmetric TAGs containing SCFAs and MCFAs—would appear in pre-digested *sn*-1,2/2,3 DAG forms in the next (ileal) stage of digestion, which PL can now cope with more easily. Moreover, the FFAs produced during gastric digestion, due to their emulsifying ability can further improve PL’s access to TAGs, thus improving the efficiency of fat digestion in the duodenum [17]. This synergistic effect of the two enzymes is clearly demonstrated in lipid digestion of substrates with substantial quantity of SCFAs and MCFAs, such as cream. Finally, it should be also emphasized that the observed variation of the lipolytic efficiency of GL and PL towards different TAGs can also be attributed to differences in the interfacial microenvironment of the enzymes. This aspect can be of special importance during real meal digestion processes, when a number of uncontrolled interfacial interactions may occur [66].

### 3.3. Effect of GSP and BTB on Lipid Digestibility

Two different types of fat-free foods with bioactive constituents with proven lipase inhibitory effects, namely grape seed powder (GSP) and black tea brew (BTB), were selected to investigate the characteristics of their effects on the digestion of milk fat (cream) and baked beef fat. First, effective treatment levels of GSP and BTB were determined by performing dose–response experiments on cream as sample test foods. The effective dosage was further tested on BB for evaluation of substrate-specific effects.

#### 3.3.1. Dose and Substrate Dependency

**Figure 3:**
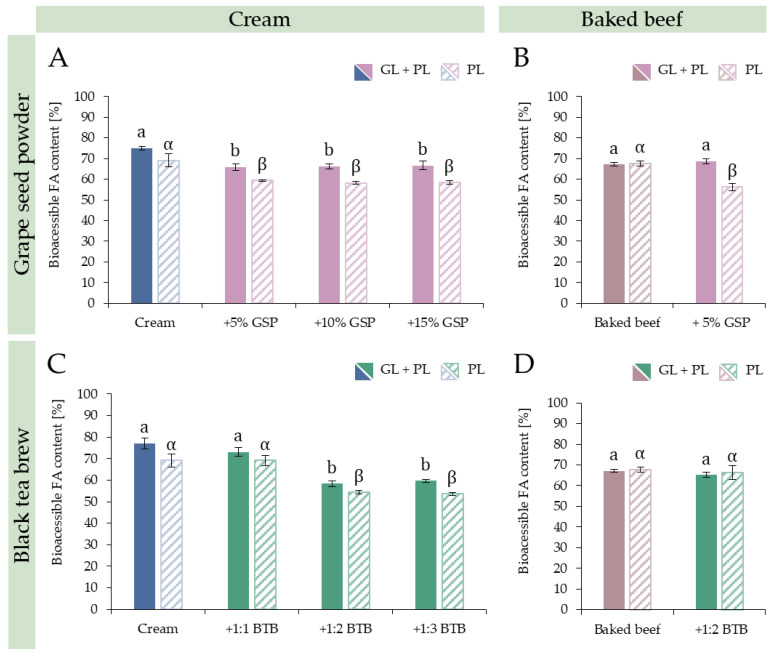
Dose dependency tests with cream test food (**A**,**C**) and effect of foods with bioactive constituents on baked beef (**B**,**D**) in the most effective ratio. (**A**,**B**): effect of grape seed powder (GSP); (**C**,**D**): effect of black tea brew (BTB). Significant difference within groups is marked with Latin letters (GL + PL) or with Greek letters (PL) (*p* < 0.05).

To evaluate the required effective dosage of the selected foods with bioactive constituents, first, co-digestion experiments with cream test food were conducted at three levels. In the case of GSP, which was available as a powder, it was tested in small quantities, namely in 5, 10 and 15 *w*/*w*% relative to the weight of cream. As Figure 3A shows, a significant decrease was observed at the first level (5% GSP) and the further addition of GSP did not result in a decrease in lipid digestibility. The same was noticed in both GL + PL and PL digestion simulations, i.e., the lowest tested level already resulted a significant effect in cream. The observed decrease in lipolysis in PL digestions indicates that the dispreference of PL to digest TAGs containing SCFAs and MCFAs was presumably further enhanced. At the same time, the results of the GL + PL digestions suggest that GSP had an impact on GL; thus, the efficacy of the lipolysis of TAGs containing SCFAs and MCFAs was decreased, i.e., these TAGs continue to reach the duodenum. Their lipolysis will not be completed in the small intestine either due to the dispreference shown by PL towards such substrates, and moreover, because this dispreference was enhanced as a result of GSP.

The 5% GSP treatment was also tried on baked beef in further co-digestion experiments (Figure 3B). Interestingly, addition of GSP only resulted in decreased lipid digestibility by (12%; *p* < 0.001) when the digestion simulation was carried out with PL protocol. In the GL + PL digestion, no change was observed (*p* = 0.316). This shows that, in the case of a less marked presence of TAGs containing SCFA and MCFA, the decrease in gross lipolysis was less significant. 

The effect of BTB was also tested with cream first. Diluted aqueous extract of a selected black tea cultivar with high tannin content was mixed with cream before digestion. In this case, cream to tea brew weight ratios of 1:1, 1:2 and 1:3 were tested (Figure 3C). Maximum lipolysis inhibitory effect of BTB was reached at the 1:2 level and a further addition did not increase the effect. The observed degree of lipolysis inhibition did not differ between the GL + PL and PL digestion simulations, 23% and 22% (*p* = 0.067) decrease in overall lipid digestibility was measured, respectively. Similarly to the experiments observed with GSP, no effect of BTB on overall lipid digestibility was observed with baked beef as a test food, when the same BTB to fat ratio was tested, which seemed effective for significant lipolysis inhibition in the case of the 1 cream: 2 tea experiment (Figure 3D). This result clearly suggests that differences in the fat source, i.e., the type and form of substrates for digestive lipase enzymes had a marked influence on the impact of the same fat-free test food on lipolysis. Most probably, the difference in FA profile, TAG structure and other matrix specific characteristics of the consumed food not only influences lipid digestibility of fat from different foods, but also influenced the lipolysis inhibitory effects of the fat-free test foods when consumed together with substrate test foods containing different types of fat sources. Lipase inhibitory properties of black tea are often attributed to theaflavins and it was also speculated that theaflavins bind to both the free enzyme as well as the enzyme-substrate complex, and likely at a site other than the substrate-binding site [48]. However, our above observation indicates that the lipolysis inhibitory property of black tea is more complex under physiologically more realistic conditions, and it can be sensitively modulated by the microstructure of the food [67].

#### 3.3.2. Fatty Acid Specific Effects: Reduced Digestibility of SCFAs and MCFAs

**Figure 4:**
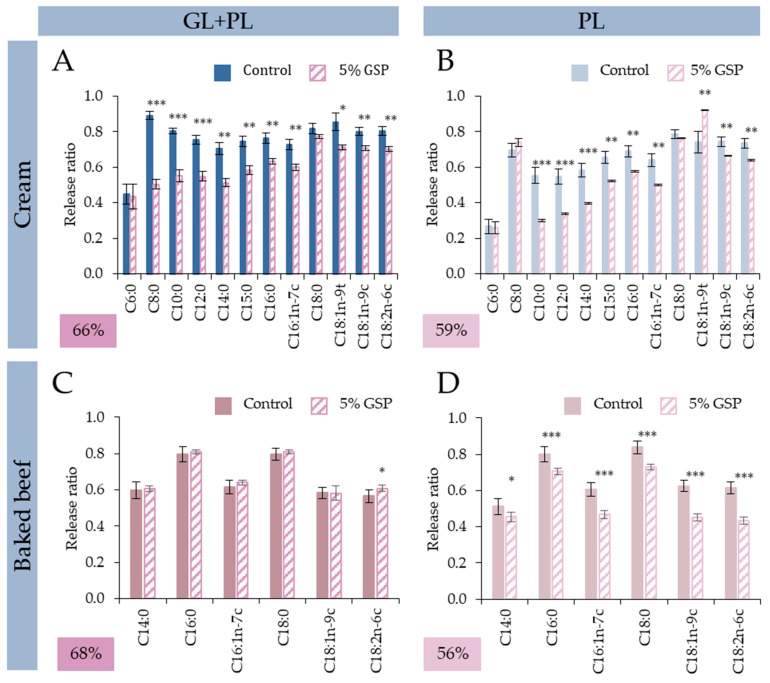
Effect of 5 w% of grape seed powder on release ratio of fatty acids. (**A**,**B**): cream test food in digestion simulation with GL + PL and PL, respectively; (**C**,**D**): baked beef test food in digestion simulation with GL + PL and PL, respectively. Asterisk labels (*) show significant difference between release ratio of individual fatty acids after GL + PL and PL digestions (* *p* < 0.05; ** *p* < 0.01; *** *p* < 0.001).

**Figure 5:**
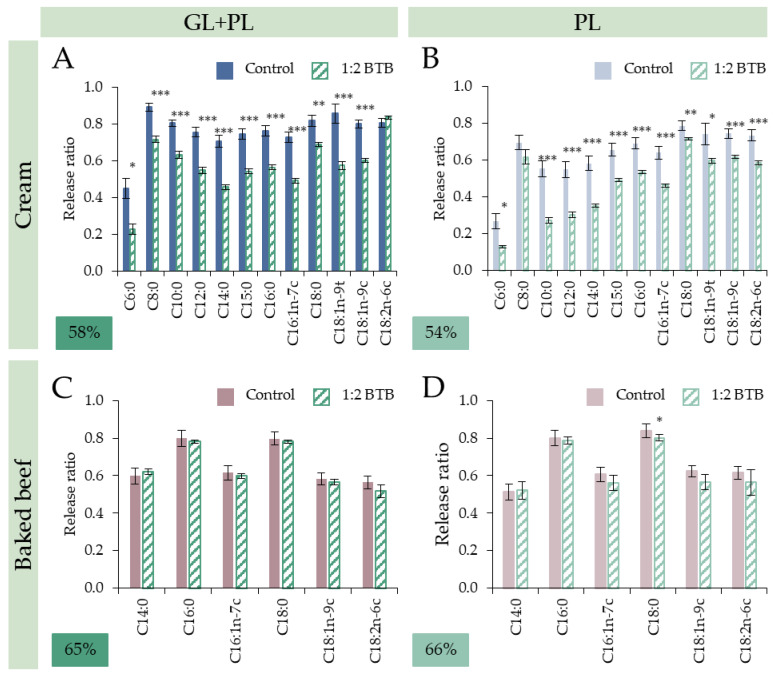
Effect of black tea brew (1:2 ratio) on release ratio of fatty acids. (**A**,**B**): cream test food in digestion simulation with GL + PL and PL, respectively; (**C**,**D**): baked beef test food in digestion simulation with GL + PL and PL, respectively. Asterisk labels (*) show significant difference between release ratio of individual fatty acids after GL + PL and PL digestions (* *p* < 0.05; ** *p* < 0.01; *** *p* < 0.001).

Our results showed that the exerted lipolysis inhibitory effects of the tested GSP and BTB were substrate-dependent, i.e., the given amount that was sufficient to restrict cream lipid digestion was not enough to provide the same impact for baked beef test food with identical lipid concentration. To further study the impact of fat-free test foods, FA-specific analysis was conducted (Figure 4 and Figure 5).

In the case of GSP and BTB treatments, a similar trend regarding the changes in FA profile could be observed for cream as a substrate test food. Co-digestion with GSP and BTB primarily affected the MCFAs. In GL + PL digestion simulations with GSP (Figure 4A), the release of SCFAs and MCFAs were reduced more drastically than the release of LCFAs. Namely, the digestibility of SCFAs and MCFAs (C8:0 C15:0) reduced in the range of 43–22% and the release of LCFAs were reduced between 5 and 18%, compared to the control digestions. In the case of BTB (Figure 5A), the inhibitory effect in digestion simulation with both enzymes were more evened out, since the reduction in the release of all FAs was between 16 and 50%; however, the average reduction was 5% higher for SCFAs and MCFAs. Moreover, the impact of both foods seemed to be more pronounced on the pancreatic lipase activity. After PL only digestions (Figure 4B and Figure 5B), the addition of both GSP and BTB resulted in a decreased release of SCFAs and MCFAs. With GSP, RR of C10:0–C15:0 decreased by 46–32% compared to average 7% decrease of longer FAs, and with BTB, RR of C6:0 and C10:0–C15:0 decreased by 52% and 51–25%, respectively, compared to the average 19% of longer FAs.

Interestingly one exception was found for BB, where lipolysis restriction was observed. Namely, when the 5% GSP was added and when only PL was used (Figure 3B). The observation could be attributed to the difference in the extent of the effect between the two fat-free test foods on PL, i.e., GSP had a higher effect on only PL than GL + PL and BTB did not. This effect was not uniform on all FAs (Figure 4D). The RR decrease was higher on the only MCFA (C14:0: 25%) and on the unsaturated FAs (C16:1n-7c: 27%, C18:1n-9c: 22% and C18:2n-6c: 29%) than on the other two, saturated FAs (C16:0: 13% and C18:0: 10%). This also highlights the specific non-preference of PL on the asymmetric TAGs, since the TAGs containing unsaturated FAs were also from this type of TAG (Figure 1).

## 4. Conclusions

In conclusion to the digestion simulation experiments carried out with two selected substrate test foods (cream and baked beef) having markedly different FA profiles, we postulate that TAGs containing SCFAs and MCFAs are non-preferred substrate types for pancreatic lipase (PL) and, consequently, PL alone is rather ineffective in the digestion of such TAGs containing SCFAs and MCFAs. However, this specificity was not characteristic for gastric lipase (GL). This effect was prominent in milk fat containing products such as the analyzed cream. The results also showed that when there was no marked diversity in FA composition of the consumed fat source and, consequently, heterogeneity in the TAG structure was limited, the contribution of GL could be negligible such as in the case of baked beef. We also found that the difference in FA profile, TAG structure and other matrix specific characteristics of the consumed food influenced the lipolysis inhibitory effects of the same fat-free food containing bioactive constituents, when consumed together with foods containing different types of fat sources.

Our findings together suggest that the lipolysis inhibitory effect of both GSP and BTB primarily affected the lipolysis of short and medium-chain FAs. The reason behind this can be explained by the hypothesis that the dispreference of PL to digest TAGs containing SCFAs and MCFAs was presumably further enhanced as a result of treatment with foods such as GSP or BTB. At the same time, we postulate that GL was also restricted to some extent and could not perform the pre-digestion of SCFA- and MCFA-containing TAGs efficiently either. It should be noted, however, that the reasons for the dispreference of PL towards TAGs containing SCFA and MCFA and the increase in dispreference when these lipids were consumed in a meal along with GSP or BTB was unclear.

Another interesting conclusion derived from this in vitro preliminary study is that the two different foods with bioactive constituents showed lipolysis-inhibiting effects with the same characteristics. That is, both GSP and BTB had a significant effect in the case of cream, while they proved to be ineffective in restricting the digestion of fat from baked beef. This finding highlights the importance of the characteristics of the fat source and the food matrix as key determinants in the observed lipolysis inhibitory effect of GSP and BTB. This observation also suggests that in future studies, the impact of various foods—rich in substances with proven lipase-inhibiting effect—on lipid digestion—should be ideally investigated with different types of real foods as substrates, which might specifically influence their presumed effects on lipolysis.

## Data Availability

Not applicable.
